# Characterising routes of H5N1 and H7N9 spread in China using Bayesian phylogeographical analysis

**DOI:** 10.1038/s41426-018-0185-z

**Published:** 2018-11-21

**Authors:** Chau M. Bui, Dillon C. Adam, Edwin Njoto, Matthew Scotch, C. Raina MacIntyre

**Affiliations:** 1 University of New South Wales (UNSW), Sydney, NSW Australia; 2 Arizona State University (ASU), Tempe, AZ USA

## Abstract

Avian influenza H5N1 subtype has caused a global public health concern due to its high pathogenicity in poultry and high case fatality rates in humans. The recently emerged H7N9 is a growing pandemic risk due to its sustained high rates of human infections, and recently acquired high pathogenicity in poultry. Here, we used Bayesian phylogeography on 265 H5N1 and 371 H7N9 haemagglutinin sequences isolated from humans, animals and the environment, to identify and compare migration patterns and factors predictive of H5N1 and H7N9 diffusion rates in China. H7N9 diffusion dynamics and predictor contributions differ from H5N1. Key determinants of spatial diffusion included: proximity between locations (for H5N1 and H7N9), and lower rural population densities (H5N1 only). For H7N9, additional predictors included low avian influenza vaccination rates, low percentage of nature reserves and high humidity levels. For both H5N1 and H7N9, we found viral migration rates from Guangdong to Guangxi and Guangdong to Hunan were highly supported transmission routes (Bayes Factor > 30). We show fundamental differences in wide-scale transmission dynamics between H5N1 and H7N9. Importantly, this indicates that avian influenza initiatives designed to control H5N1 may not be sufficient for controlling the H7N9 epidemic. We suggest control and prevention activities to specifically target poultry transportation networks between Central, Pan-Pearl River Delta and South-West regions.

## Introduction

Avian influenza (AI) is a threat to both animal and human health in China. In the past, H5N1 strains have caused global concern due to their high pathogenicity in poultry and high reported case fatality rates in humans. Since 2013 four novel zoonotic strains of AI have emerged from Asia^1^. Of these, the H7N9 subtype has a high pandemic potential^2^. The H7N9 subtype was first reported in humans in 2013 and caused large outbreaks in humans every winter season in China^3^. Over 1500 H7N9 cases have been reported over the past 5 years, whereas only 860 H5N1 cases have been reported over the past 20 years^3,4^. Recent studies comparing the epidemiology of H5N1 and H7N9 show that human H7N9 cases report lower levels of contact with sick or dead birds^5^, detection rates in poultry are lower for H7N9^6^, and the geographic distribution of H7N9 outbreaks (in the first four waves) have been much more limited to south-eastern regions of China^7^ (including Guangxi, Guangdong, Hunan, Hubei, Jiangxi, Fujian, Zhejiang, Anhui, Shanghai, Jiangsu, Henan and Shandong administrative regions—i.e. Eastern and South Central traditional regions as defined in Lu et al.^8^).

Genetic sequence data can be used to model the evolutionary relationships between virus samples and help to explain epidemiological patterns and uncover processes of transmission. For example, Shi et al.^9^ used phylogenetic analysis to show human infections of H7N9 originated from poultry—they found high homology between H7N9 isolated from humans with those isolated from live poultry markets. In rapidly evolving pathogens, such as influenza viruses, evolution occurring over time can occur concurrently with geographic spread over time—by incorporating evolutionary dynamics with temporal and spatial attributes of individual sequences, spatial phylodynamic processes can be described^10,11^. For example, Lam et al.^12^ used phylogeographic analyses to infer the origins and dispersal patterns of three separate clades of H7N9. They found one clade spread outwards from Zhejiang province (Yangtze River delta region), another circulated extensively within the Pearl River Delta, while a separate clade was widespread across eastern China. Many other studies have utilised phylogeographic approaches to characterise the geographic dispersal of H5N1 ^13–17^ and H7N9 ^18–20^ viruses.

The direction and speed of AI virus spread is determined by a number of interdependent factors such as wild bird migration, poultry trading routes, farming and livestock practices, human population density, avian population density, mixing between humans and birds, and climate^21^. The generalised linear model (GLM) is a recently developed technique that incorporates these factors into the phylogeographic network model and measures their effect on the model^22^. GLM analyses have been used to estimate the migratory patterns of influenza A H7N7 in the Netherlands^23^, quantify economic-agricultural predictors of AI spread in China^8^, identify air travel and global mobility as key drivers of human H3N2 diffusion^22^ and demonstrate that global live swine trade strongly predicts spatial dissemination of influenza A viruses in swine^24^. The GLM has also been used to analyse H5N1 specifically. Beard et al.^25^ used the GLM approach to confirm avian population density as a major contributor to the viral diffusion of H5N1 clade 2.2.1.1 in Egypt. Trovao et al.^26^ quantified predictors of H5N1 spread within Asia and Russia using a modified GLM that incorporated random effects to allow for spatial transmission patterns that deviate from regular distance-based dispersal dynamics. To the authors’ knowledge, this is the first study to use a GLM approach in the context of H7N9.

In this study we aimed to construct independent discrete trait Bayesian phylogeographic models with extended GLM analysis to characterise routes of H7N9 and H5N1 diffusion and quantify the contribution of potential drivers of viral spread. Understanding the differences and similarities of H7N9 and H5N1 diffusion across China can be useful for planning targeted control and prevention strategies.

## Results

**Fig. 1:**
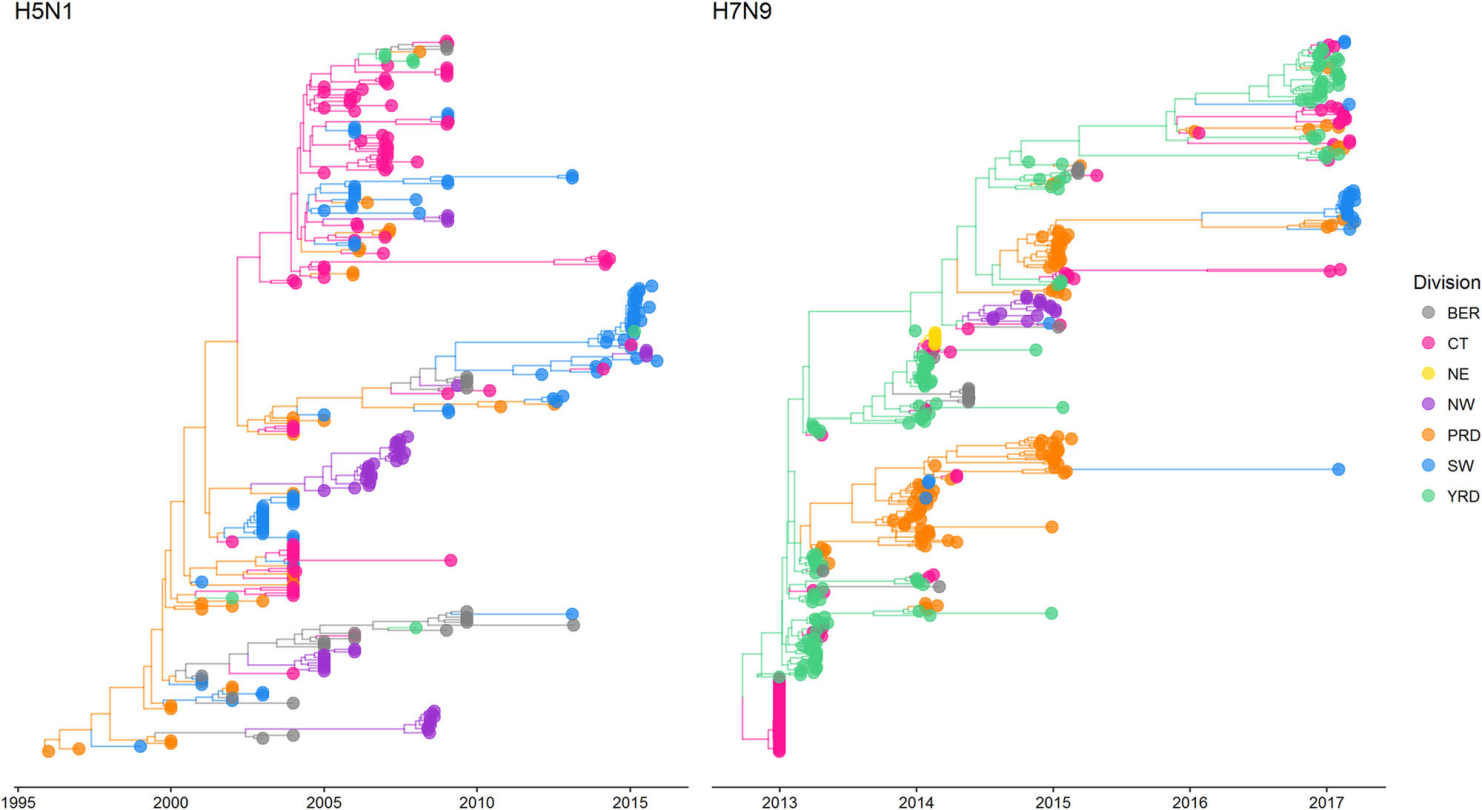
Phylogeography models of H5N1 and H7N9. Bayesian MCC phylogeneies and between-region diffusion networks on HA gene segments of H5N1 (left panel) and H7N9 (right panel) in China. The sequences are classified according to their location grouped by economic division. Bohai Economic Rim (BER): Beijing, Hebei, Shandong; Central (CT): Anhui, Henan, Hubei, Hunan, Jiangxi, Shanxi; North-east (NE): Heilongjiang, Jilin, Liaoning; North-west (NW): Gansu, Ningxia, Qinghai, Shaanxi, Xinjiang; Pan-Pearl River Delta (PRD): Fujian, Guangdong; South-west (SW): Guangxi, Guizhou, Sichuan, Tibet, Yunnan, Chongqing; Yangtze-River Delta (YRD): Jiangsu, Shanghai, Zhejiang. Trees have been scaled according to taxa dates (representing sample collection date)

In Fig. 1 and Figure S1, we show the maximum clade credibility (MCC) trees for H5N1 and H7N9. To improve visualisation, Fig. 1 shows MCC trees with locations grouped by economic division (grouping administrative regions into economic divisions was conducted after analyses were completed; hence, grouping had no effect on the evolutionary trees themselves—Figure S1 shows MCC trees prior to grouping locations by economic division). Grouping by economic zones was chosen as the preferred grouping method, based on similar GLM analyses conducted by Lu et al.^8^ which used this grouping method (among others), and found it amenable to demonstrating avian influenza diffusion patterns. In Supplementary Files 1−2, we show animated MCC trees of the H5N1 and H7N9 migration processes, and these can be visualised using Google Earth. We show that H5N1 and H7N9 evolutionary relationships exhibit stronger clustering by administrative and economic regions.

For H5N1, separate lineages appeared to have been circulating at the same time over a spread of regions (particularly central, south-western, Pearl River Delta regions and North-western regions). Guangdong notably plays an important role in seeding viral dissemination. North-western regions do not seem to play a role for further dissemination of virus in China. The most recently sampled sequences (sampled from 2014 to 2015) appear to cluster in the South-western regions, with evidence of migration to North-western, Yangtze River Delta and Central regions.

For H7N9, geographic dissemination appears more concentrated to south-eastern regions of China compared to H5N1. The Yangtze River Delta region was host to a wide range of early ancestral H7N9 lineages which then went on to circulate in the Pearl River Delta region. Yangtze River Delta and Pearl River Delta regions harbour most of the viral transmission, with only occasional migrations occurring to other regions of China. The most recent sequences (sampled between 2016 and 2017 and representing the fifth wave of H7N9 outbreaks) predominantly form two separate clades: one is mostly circulating in the Yangtze River Delta as well as Central and Pearl River Delta regions, and the other is mostly circulating in South-western regions of China.

**Fig. 2:**
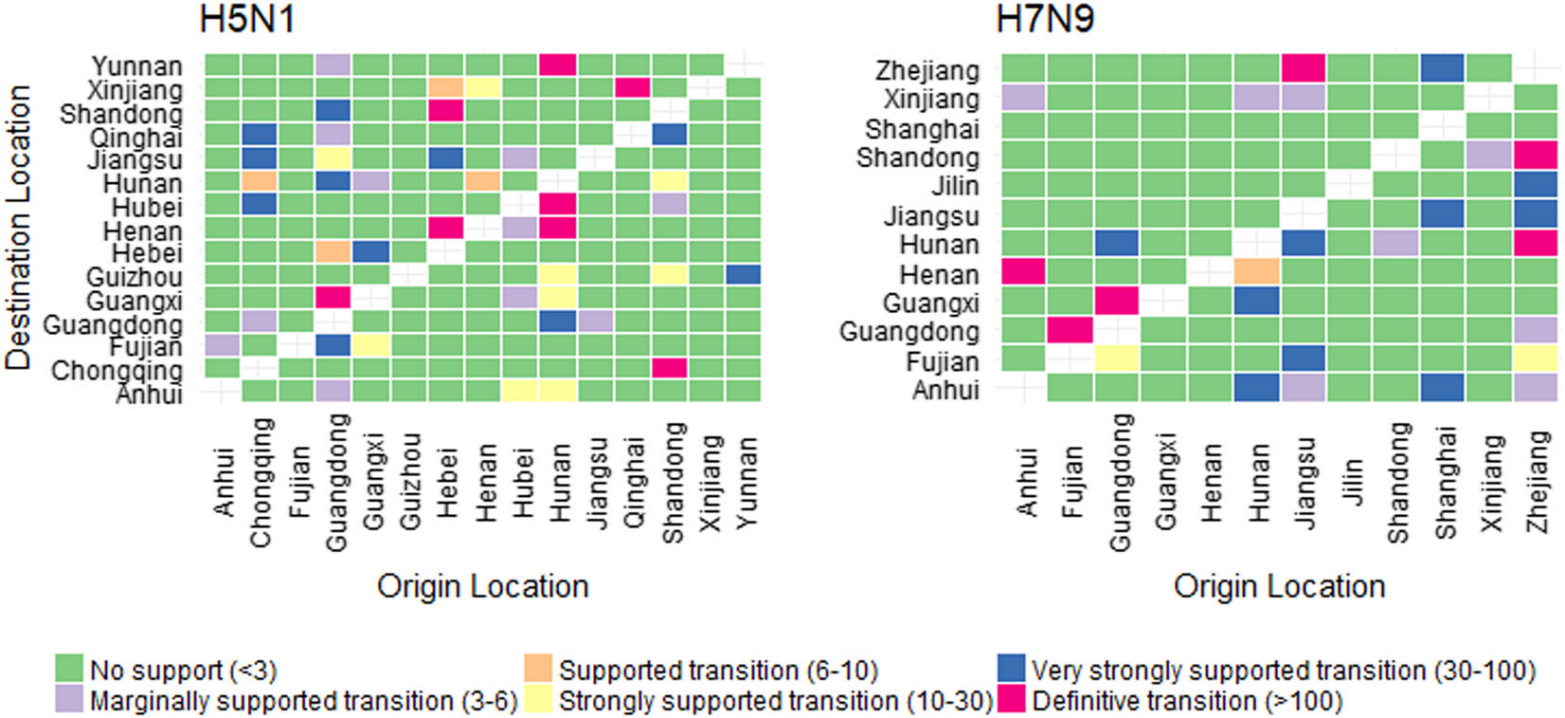
Level of Bayes Factor support for each transmission route. The left and right panels display the level of Bayes Factor (BF) support for each of the transmission routes considered for H5N1 and H7N9 analyses respectively. The *x*-axis represents the origin location and the *y*-axis represents the destination. Level of BF support is coloured according to classifications described in Table S1.

We quantified patterns of H5N1 and H7N9 spatial diffusion under a Bayesian stochastic search variable selection (BSSVS) procedure. In Fig. 2, we show graphs of the level of support for each of the transitions analysed, using Bayes Factor (BF) cut-offs described in Lemey et al.^15^, shown in Table S1. We found only two highly (definitive and very strongly) supported transitions were common to both H5N1 and H7N9: Guangdong to Guangxi (definitive transition, BF > 100), and Guangdong to Hunan (very strongly supportive transition, BF 30–100). For H5N1, most highly supported transitions originated from either Guangdong or Hunan whereas for H7N9, most highly supported transitions originated from Zhejiang.

### GLM analysis

**Fig. 3:**
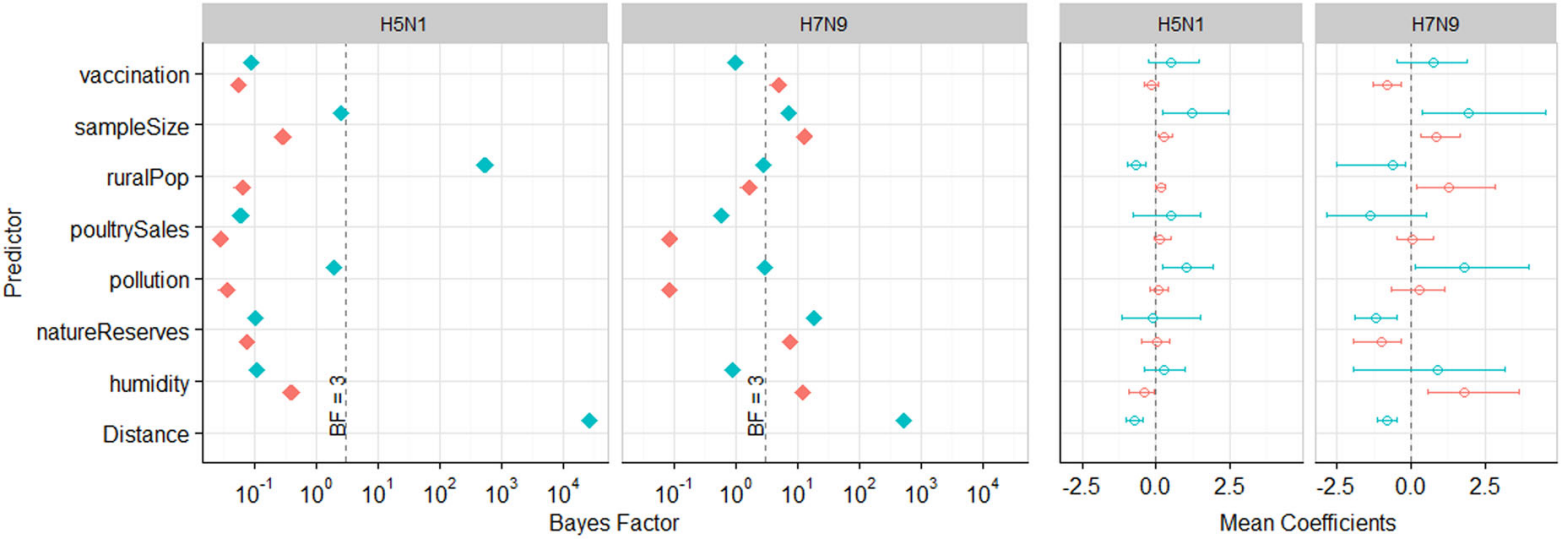
Generalised linear model. From left to right, the two panels show (i) Bayes Factor (BF), and (ii) Mean Coefficients (meanCeffects) and their 95% highest posterior credible intervals. Plots for H5N1 and H7N9 are displayed side by side. For all predictors excluding Distance, green and orange colours represent origin and destination locations respectively. In the BF plots, the dashed line indicates BF = 3. In the meanCeffects plot, the dashed line indicates 0. Note BF results are displayed on a log10 scale

In Fig. 3 (and Tables S2-3), we show results of the GLM analysis (we used BF cut-offs described in Lemey et al.^15^, shown in Table S1). For H5N1, we found that distance between two locations and rural population at the origin location had definitive support (BF > 100) for inclusion into the model. Both predictors were found to have a negative mean coefficient—H5N1 viral dissemination is associated with a decrease in distance between two locations, and a lower (rather than higher) rural population density. We did not find any other predictor to have support (BF > 3) for H5N1. For H7N9, only distance had definitive support (BF > 100), and like H5N1, showed a negative relationship (implying these factors have a protective effect) to viral transmission. We found six other predictors to have marginal (BF > 3) to strong (BF > 30) support, including: vaccination rate (at the destination), sampling size (at both origin and destination locations), nature reserves (at both origin and destination locations), and the average relative humidity of major cities at the destination location.

## Discussion

Zoonotic avian influenza (AI) poses a major risk to both human health and poultry production industries in China. Our study used H5N1 and H7N9 sequence data to explore transmission dynamics and identify potential drivers of viral spread. Using discrete state Bayesian phylogeography we demonstrate that H5N1 and H7N9 have different spatial patterns and drivers of diffusion. For H5N1, we found Guangdong is primarily associated with seeding viral dissemination while for H7N9 we found two distinct groups circulating predominantly in the Pearl River Delta and Yangtze River Delta regions. Determinants of viral diffusion differed markedly between H5N1 and H7N9: proximity between locations was found to be a strong predictor for H5N1 and H7N9; however, low rural population density was only found to be a strong predictor for H5N1, and for H7N9, low avian influenza vaccination rates at destination locations, low percentage of nature reserves and high humidity levels at destination locations were drivers of viral diffusion.

There is a general trend for AI outbreaks and spreading dynamics of AI viruses to concentrate in south-eastern regions. Previous phylogeography studies of multiple AI subtypes have shown that spread primarily originates from Yangtze River Delta, and South Central regions towards the east coast areas^8^. With regards to H9N2, Guangdong province and Jiangsu province were found to be primary and secondary sources of seeding outbreaks^27^. More detailed analyses of virus migrations show that movement dynamics within the general south-eastern region of China are complex, and these heterogeneous dynamics differ for each subtype.

Together, the diffusion and GLM results strongly suggest that there are fundamental differences in large-scale transmission dynamics between the two subtypes that reiterates the findings of our previous study exploring static spatial distributions of H5N1 and H7N9^7^. Temporality may explain this difference—improvements in AI diagnostic capabilities, reporting systems, as well as improvements in AI awareness and control within the poultry industry have likely changed how AI disseminated across China over time. However, from a close examination of the dynamic maps of H5N1 and H7N9 spread, there is little overlap in the speed or direction of each subtypes’ movement. We recommend further examination of AI transmission dynamics at more specific, higher-scale spatial resolutions—this will allow for a stronger level of support of predictor contributions. Such analyses can only be conducted if location metadata is annotated at the level of secondary administrative regions or higher and we recommend there be effort made to ensure detailed recording of sequence metadata in genetic data repositories.

It is known the three strong economic regions of China: the Bohai Economic Rim, Pearl River Delta and Yangtze River Delta regions are highly connected through advanced transport infrastructures that facilitate regional movement of live poultry^28,29^—our findings suggest that transportation networks between Central, Pan-Pearl River Delta and South-West regions should be considered key routes for AI dissemination.

Previous studies show H5N1 and H7N9 movement occurs mostly between regions in close proximity via poultry trading, as opposed to movement over long distances via wild bird migration^30^. The spread of H7N9 across China primarily occurs through movements of domestic poultry (as opposed to seasonal wild migratory bird migrations). H7N9 has only very rarely been found in wild birds and is only occasionally identified in live poultry^6^. H5N1, on the other hand, has been shown to have spread through seasonal wild bird migratory pathways; however, our analyses suggest this mechanism does not play a significant role in dissemination of disease^30–34^.

The production and marketing of poultry in China consists of a heterogenous network, made up of traditional farming mixed with commercial operations and range considerably in size^35^. Live bird movements along broiler and layer poultry supply chains (breeding, hatching, fattening, feeding, slaughter, wholesale and retail markets, consumption, and exporting) is considered to be too complex to be characterised on both national and provincial scales^35^. Our phylogeographic networks for H5N1 and H7N9 are likely to be indicative of poultry movements across China, hence could be a potential data source for future network modelling research.

AI remains an important global disease affecting both human and animal populations. H5N1 has caused over 800 human cases over two decades of circulation, whereas the novel H7N9 has caused over 1500 human cases over a 5-year period^4^. In the winter season of 2016–2017, there was a significant increase in the number of H7N9 cases reported during the fifth wave compared to all the other waves combined^36^. An increased number of H7N9 cases were found in rural and semi-urban regions in China. Some of these regions had not previously reported H7N9 prior to the fifth wave. In the fourth and fifth waves, the proportion of H7N9 cases from semi-urban and rural residents has grown, comprising up to about 60% of the cases; a steady rise from 39% reported during the first wave^36^. The increased number of human infections appears to be associated with wider geographic spread and higher prevalence of Asian H7N9 viruses among poultry rather than any increased incidence of poultry-to-human transmission^36^. In Zhejiang, the increase in H7N9 cases in the fifth wave is attributed to spread to areas where live bird markets were not permanently closed^37^. There is more dispersed geographic incidence of human cases in the fifth wave compared to highly geographically clustered cases in the South-Eastern seaboard of China in previous waves. In our phylogeography model, we find that during the fifth wave there were new H7N9 transmission routes occurring from Jiangsu to Guangxi, and from Hunan to Henan and Guangxi—however compared to H5N1, this spread is not as geographically extensive. This may be due to a paucity of sequence data available from the fifth wave outbreaks. This may limit the interpretation of more recent diffusion in our model. It will be useful for future research to explore the phylodynamics of H7N9 fifth wave cases when they become available.

This study is subject to certain limitations. Sequence samples of H5N1 and H7N9 are unlikely to be representative of every AI clade across each discrete region. There are large discrepancies in the type (passive or active) and quality of surveillance between provinces and over time. It is likely that viruses sampled here are concentrated in high-risk areas potentially resulting in sampling bias and therefore inaccurate ancestral reconstruction processes^38^. We however, attempted to characterise these biases in terms of spatial and temporal distributions—presented in detail in Supplementary File 3. The proportion of sequence data over time generally reflects that of disease incidence, with the exception of H5N1 prior to 2004, as incidence data were not available prior to 2004 for this subtype. For H7N9, the ratio of sequences to incidence data is much smaller due to the large number of H7N9 human cases. There were much greater discrepancies between the geographic distribution of sequences and disease incidence data for both subtypes. Compared to H5N1, there are much larger discrepancies for H7N9.

We also attempted to reduce sampling biases by downsampling in regions where there were too many samples (*n* > 50), as previous studies have done^39,40^. The geographic and temporal scale of our GLM analysis is relative large (for example, our H5N1 analysis spanned around 20 years), in addition the areas of primary administrative regions in China are very diverse (for example, Shanghai is 6340 km^2^ whilst Xinjiang is 1,664,900 km^2^). Predictor contributions at such a broad scale of analysis may lose their statistical significance at smaller scales, and there are many complexities in how these predictors contribute to virus evolution which cannot be accounted for. We also excluded regions for which there were few sequences; however, we acknowledge that regions with small sequence sample size may sometimes provide very significant genetic differences. A descriptive assessment of sampling biases and their influence of tree topology is presented in Supplementary File 3, which show our method of downsampling did not affect tree topology. Future research should statistically evaluate the influence of sampling biases on tree topology. In addition, we do not account for changes in predictors over time. In future studies for example, relationships between virus evolution and vaccination rates may be more evident with a time-series approach^41^ rather than a geographic approach.

As H7N9 continues to cause human infections, and other zoonotic AI continue to emerge, it becomes more important for public health professionals to exploit findings from evolutionary and spatiotemporal analyses to develop more efficient control and prevention measures. We recommend future research examine predictor contributions at higher spatial resolutions to identify whether predictor support remains the same for specific localities—this can also more directly inform public health action. We urge researchers, data analyst, epidemiologists, policy makers, field surveillance officers to report more specific location metadata in genetic data repositories to allow for such analyses.

## Materials and methods

### Sequence collection, selection and alignment

We downloaded a total of 3305 full-length haemagglutinin (HA) genes of H5N1 viruses and 1363 full-length HA genes of H7N9 viruses from GISAID (from 1996 to 2017)^42^. We included sequences only from Mainland China with discrete location metadata (to at least primary administrative regions of province, municipalities or autonomous regions). We additionally excluded regions where only a small number of sequences were available (H5N1 *n* < 7; H7N9 *n* < 4). We excluded 8 regions for H5N1 and 12 for H7N9. In total, we selected 265 sequences across 15 discrete regions for inclusion into the H5N1 study, and 371 sequences across 12 discrete regions for inclusion into the H7N9 study (see Table S4). For H7N9, we randomly down-sampled sequences from regions which had over 50 sequences (more details are available in Supplementary File 3). We aligned sequences separately for H5N1 and H7N9 using the MUSCLE plugin for Geneious 10.0.8 (Biomatters). We describe additional details of the selection procedure in Supplementary File 3.

### Construction of the discrete state phylogeography models

We produced ultrametric phylogenetic trees using a Bayesian Markov chain Monte Carlo (MCMC) approach available in BEAST v1.8.4^43^. We specified a reversible continuous-time Markov chain (CTMC) model to estimate transitioning among discrete location states throughout evolutionary history^15^. Based on other H5N1 and H7N9 phylodynamic analyses^14,18,19,41,44–50^, we specified a range of nucleotide substitution models (GTR + G(Γ4) + I and SRD06)^51^, clock models (strict and relaxed uncorrelated log normal molecular clock)^52^ and tree models (constant, exponential, Bayesian Skygrid and Bayesian Skyline) for model testing. We specified a priori mean clock rates of normal distribution with mean of 4.29E-3 and 4.09E-3 for H5N1 and H7N9 respectively as previously determined^45,53,54^. Initial root heights (of 20.5 and 4.5 years respectively for H5N1 and H7N9) were specified by obtaining mean root heights from preliminary phylogeny models which used a constant size demographic model.

For each model, we ran an MCMC for 10^8^ generations with subsampling every 10^4^ iterations. We assessed convergence of the MCMC and sufficient sampling from the posterior (effective sample size > 200) using Tracer v1.6. Model fit was assessed through log marginal likelihoods obtained through Path Sampling and Stepping Stone Sampling analysis between the prior and posterior^55,56^. We quantified patterns of H5N1 and H7N9 spatial diffusion under a BSSVS procedure.

For H5N1, we identified that the Bayesian Skygrid coalescent model, relaxed clock model and GTR nucleotide substitution model have the highest negative log likelihood scores. For H7N9, we identified that the Bayesian Skygrid coalescent model, relaxed clock model and SDR06 nucleotide substitution model have the highest negative log likelihood scores (Figure S2). For each of the above final phylogeography models, we created a MCC tree by discarding 10% burn-in from a posterior set of 10,000 trees in TreeAnnotator v1.8.3. We visualised the MCC trees using ggtree^57^. We used SpreaD3 v0.9.6^58^ to develop interactive visualisations of the dispersal process through time and to compute a BF test to assess the support for significant individual transitions between discrete geographic locations. SpreaD3 takes a rate matrix file for location states generated under the BEAST analysis using the BSSVS procedure. The BF test identifies support for non-zero transmission routes^58^. We interpreted BF results according to Lemey et al.^15^, as shown in Table S1. We used R to create plots showing results of BF tests.

### Construction of the generalised linear models (GLM)

To test the contribution of potential predictors for the CTMC transition rate matrix between locations, we used an extension of the phylogenetic diffusion model to parameterise these rates as a log-linear function of a set of predictor matrices within a GLM framework^22,59^. The GLM approach is described in detail in Lemey et al.^60^. Spatial patterns of viral diffusion are reconstructed at the same time as assessing potential contributing factors. We identified potential predictors from previous studies, and used correlation tests to create a set that achieved full rank. Similar to Lu et al.^8^, we collated anthropogenic, agricultural and environmental data from the 2013 China statistical yearbook and the 2012 China agricultural yearbook. Details are provided in Supplementary File 2 (pages 13–15**)**.

For each virus, we selected eight predictor variables (see Table 1). For our nonreversible model, we considered each predictor as an origin and destination, except for distance. We used a Python script developed by Magee et al.^39^ to log-transform, standardise, and incorporate model predictors into the phylogenetic diffusion model. For each predictor, we obtained the mean posterior probability of inclusion, BF support value, and contribution of each predictor to the log-linear rate matrix. We used R to calculate the BF as described in previous analyses^15,25^. R (available from https://www.r-project.org/) is a free language and software environment for statistical computing and graphics.

**Table 1:** Potential predictors collated for generalised linear model (GLM) analysis

Category	Predictor name	Description
Anthropogenic	ruralPop	Rural population (%)
	pollution	Sum of smoke and dust, sulphur dioxide, nitrogen oxides (10,000 tonnes)
Agricultural	poultrySales	Sales of poultry per capita rural household (kg)
	Vaccination	Average 2014 monthly H5N1 vaccination rate (%)
Environmental	natureReserves	Percentage of nature reserves in the region (%)
	humidity	Average relative humidity of major cities (%)
Sampling bias	sampleSize	Total number of sequences used in the analysis
Geographical	Distance	Distance between two locations, calculated using latitude and longitude coordinates

## Electronic supplementary material

Figure S1. Phylogeography models of H5N1 and H7N9

Figure S2. Negative log likelihood scores from path sampling (PS) and stepping stone sampling (SSS) methods

README

Supplementary File 1. Animation of the H5N1 maximum clade credibility (MCC) tree migration process

Supplementary File 2. Animation of the H7N9 maximum clade credibility (MCC) tree migration process

Supplementary File 3. Additional materials and methods

Supplementary File 4. Acknowledgement of all sources of H5N1 sequences

Supplementary File 5. Acknowledgement of all sources of H7N9 sequences

Supplementary Tables

## Data Availability

Data used in this analysis were compiled from publically available sources. We have provided a reference where a data source is mentioned. Readers may access the data through the links provided in each reference.
