# Supplementary figures and images for: Characterising routes of H5N1 and H7N9 spread in China using Bayesian phylogeographical analysis
Source: Emerg Microbes Infect. 2018 Nov 21;7:184. doi: 10.1038/s41426-018-0185-z (PMC6246557; doi:10.1038/s41426-018-0185-z)

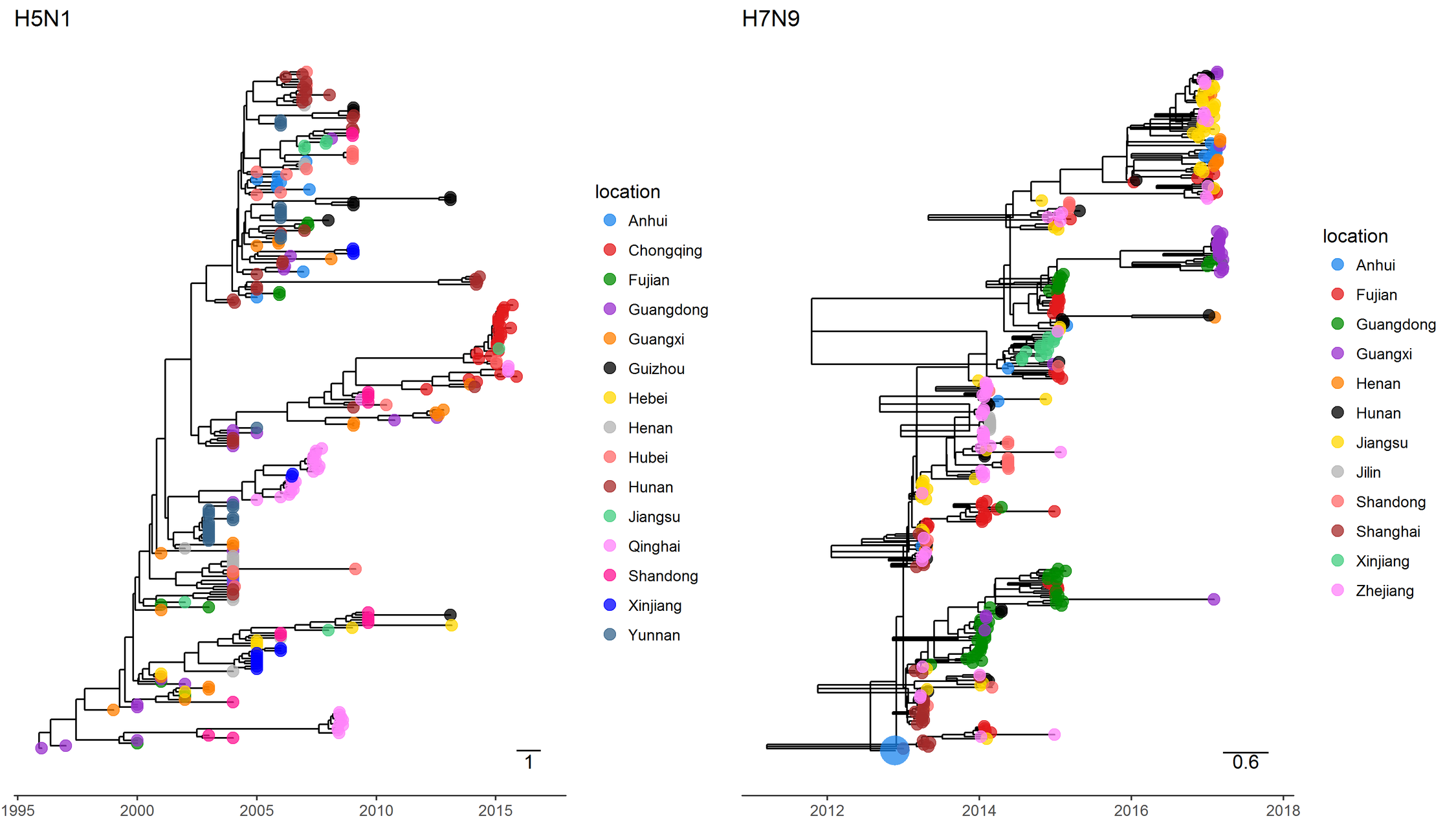

Supplement: Supplementary file 1 — Figure S1. Phylogeography models of H5N1 and H7N9 [file 41426_2018_185_MOESM1_ESM.tif]

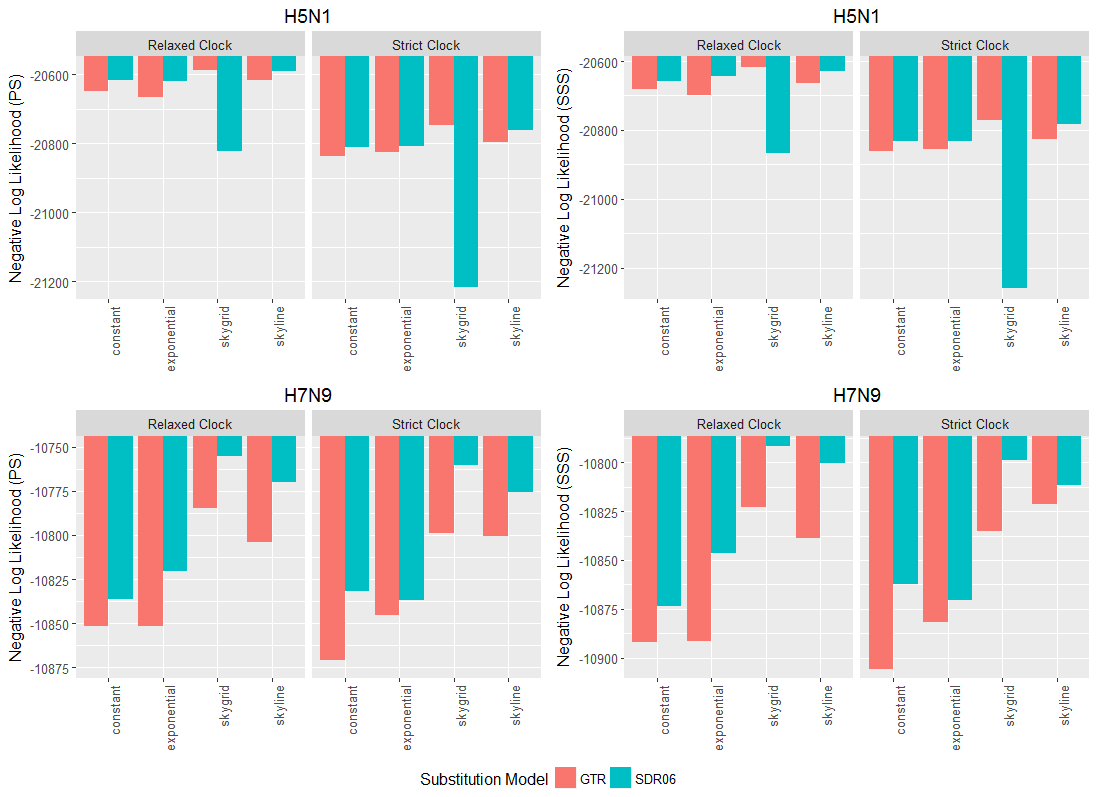

Supplement: Supplementary file 2 — Figure S2. Negative log likelihood scores from path sampling (PS) and stepping stone sampling (SSS) methods [file 41426_2018_185_MOESM2_ESM.tif]
