# Supplementary material for: Characterising routes of H5N1 and H7N9 spread in China using Bayesian phylogeographical analysis
Source: Emerg Microbes Infect. 2018 Nov 21;7:184. doi: 10.1038/s41426-018-0185-z (PMC6246557; doi:10.1038/s41426-018-0185-z)
Supplement: Supplementary file 6 — Supplementary File 3. Additional materials and methods [file 41426_2018_185_MOESM6_ESM.docx]

**Supplementary Methods**

**Assessing sampling biases incurred through excluding sequences**

Each sequence in GISAID is annotated with a description of the location and date of virus sample collection. Location metadata was used to systematically select sequences for inclusion into the study. The whole of Mainland China (approximately 9,596,960 km^2^) was selected as the study area. Mainland China is defined as all regions under the direct jurisdiction of the People's Republic of China (PRC), excluding Hong Kong and Taiwan. All autochthonous H7N9 cases have so far been isolated from Mainland China, whereas Hong Kong and Taiwan have not yet reported autochthonous human or animal H7N9 cases.

We used a systematic process to select sequence data into our study sample (see **Figure SF1**). Firstly, sequences with no usable geographic metadata were removed. Secondly, sequences outside of China were removed. Hong Kong and Taiwanese sequences were also excluded, as all autochthonous H7N9 cases have so far been isolated from Mainland China (defined as all regions under the direct jurisdiction of the People's Republic of China), whereas Hong Kong and Taiwan have not yet reported autochthonous human or animal H7N9 cases. Finally, sequences which were annotated with a spatially disaggregate location at least to the level of a primary administrative region (provinces, municipalities, autonomous regions) were chosen for inclusion into the final group.

**Figure SF1. Sequence selection flowchart.** This flowchart shows how sequences were systematically selected. The left and right panels pertain to H5N1 and H7N9 respectively.


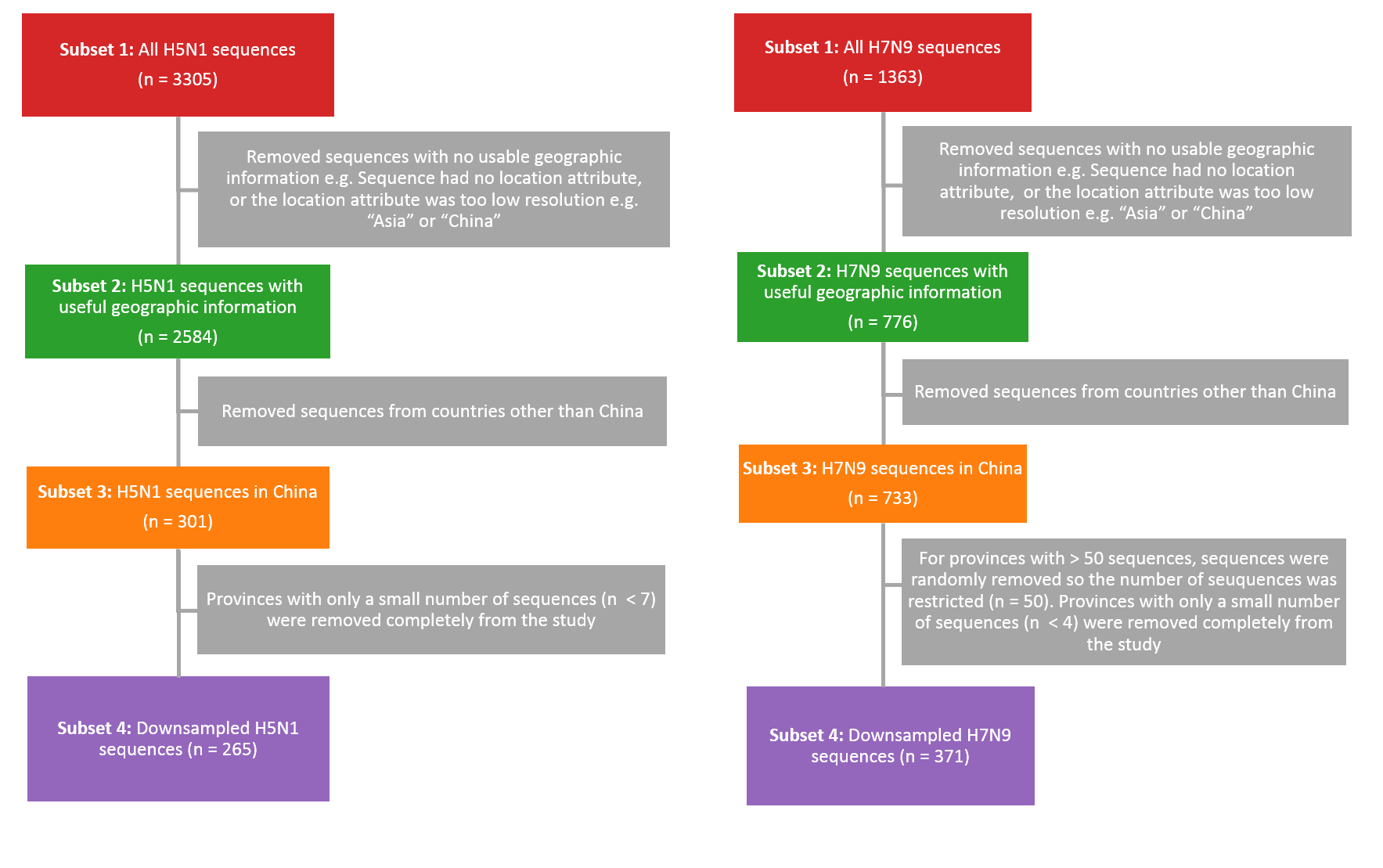


We used MEGA 7 [^1^](#_ENREF_1) to develop neighbour-joining trees. To assess whether excluding sequences produced any changes in the topology of the tree, we visually inspected each neighbour-joining tree using Phylo.io [^2^](#_ENREF_2) and compared principal coordinates using TreeSpace (λ = 0) [^3^](#_ENREF_3). For both H5N1 and H7N9, MDS analysis and visual inspection of neighbour-joining tree topologies showed that trees constructed using all four sequence subsets were not all similar to each other (see **Figure SF2**). For both H5N1 and H7N9, tree topologies were only similar between the final sequence set (subset 4) and the sequence set compiled prior to down-sampling (subset 3). We also compared the topology of final Bayesian phylogeography trees (random subsample of 100 trees and the MCC tree). For H5N1, we found that the trees developed in BEAST overlapped in “tree space” with three out of four neighbour-joining trees (*NJ subsets 1-3*).

**Assessing sampling biases incurred from H7N9 random selection**

We assessed sampling biases incurred from random selection of H7N9 by conducting individual analyses using all each of the three H7N9 sequence sets for comparison. We conducted three independent runs for each of the four analyses (one H5N1 and three H7N9), to assess for any spurious changes in parameterisation or tree topologies (indicative of insufficient sampling in the MCMC).

For H7N9, we found that the trees developed in BEAST did not overlap in “tree space” with the majority of the neighbour-joining trees, the exception being *NJ subset 4* in the second random sample of H7N9 sequences.

**Figure SF2. Evaluating similarity of tree topologies.** Panel A pertains to H5N1. Panels B, C and D pertain to H7N9 and represent the three random subsets of H7N9 sequences. The blue dots represent a subsample of 100 trees generated from the final Bayesian phylogeography analysis. The brown dots represent the MCC trees generated from the final Bayesian phylogeography analysis. The red, green, orange and purple dots represent neighbour-joining trees generated from four sequence datasets (subset 1,2,3 and 4 respectively, as described in Figure S1). Trees which are closer together in x-y distance are more topologically similar.

**
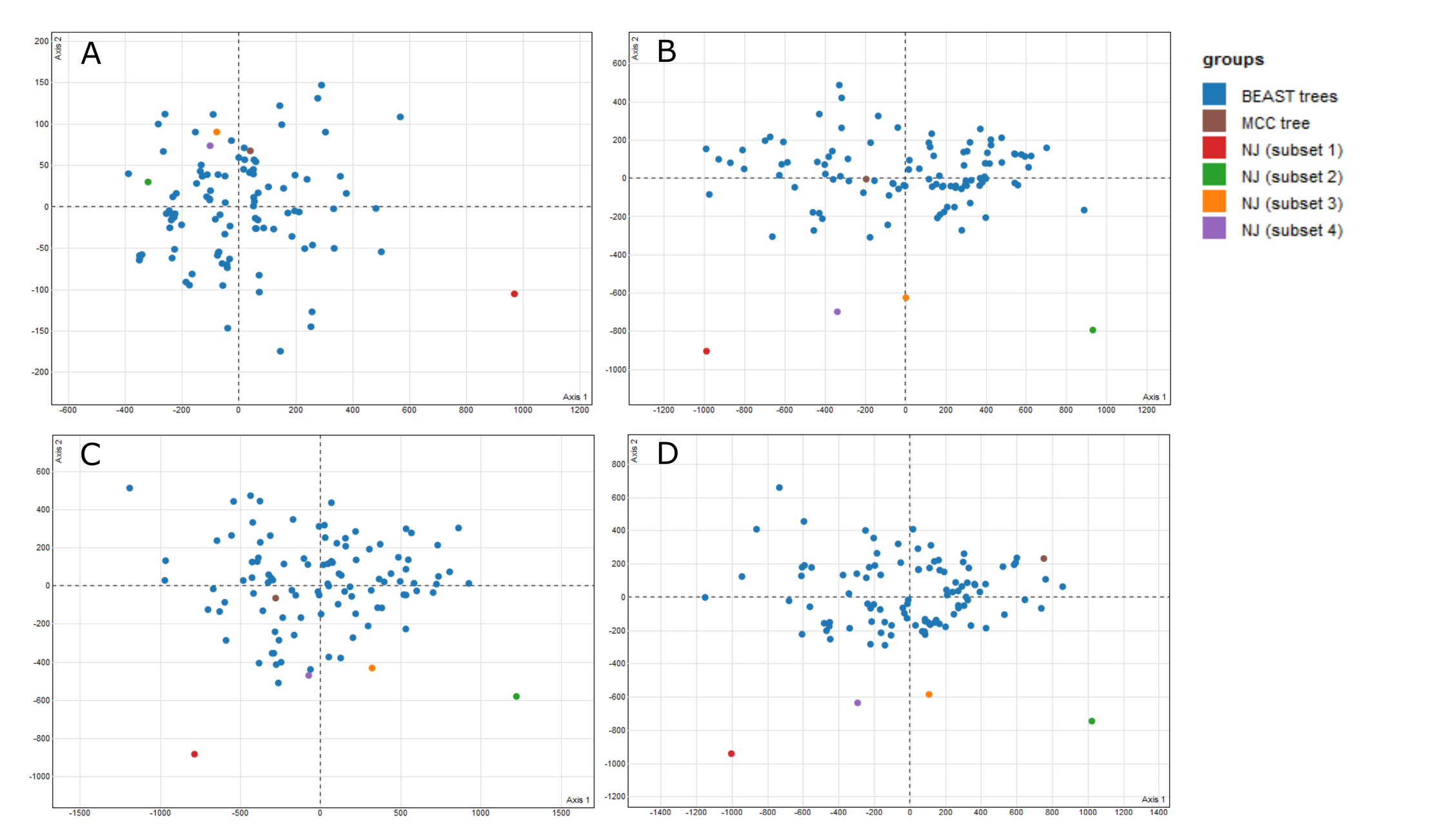
**

**Assessing representativeness of sequence data**

To assess whether our sample of sequence data was spatially and temporally representative of H5N1 and H7N9 prevalence in our study region, we compared their respective spatial and temporal distributions with human and poultry outbreak incidence data. The total number of human cases and poultry outbreaks of HPAI H5N1 and LPAI H7N9 for each primary administrative region in China was obtained from the Food and Agricultural Organization (FAO) (last accessed May 2017) [^4^](#_ENREF_4).

We visually compared the distribution of sequences and disease incidence data (human cases/poultry outbreaks) across geographical and temporal distributions **(Figures SF3-5).** We can see that the proportion of sequence data over time generally reflects that of disease incidence, with the exception of H5N1 prior to 2004, as incidence data was not available prior to 2004 for this subtype. For H7N9, the ratio of sequences to incidence data is much smaller due to the large number of H7N9 human cases. There are much greater discrepancies between the geographic distribution of sequences and disease incidence data for both subtypes. Compared to H5N1, there are much larger discrepancies for H7N9.

**Figure SF3. Comparison of sequence and outbreak data over time.** The top and bottom panels pertains to H5N1 and H7N9 respectively. Orange and green colours indicate sequence data and poultry outbreak/human case data respectively. For H5N1, data for poultry outbreak/human case data was not available prior to 2004.

**
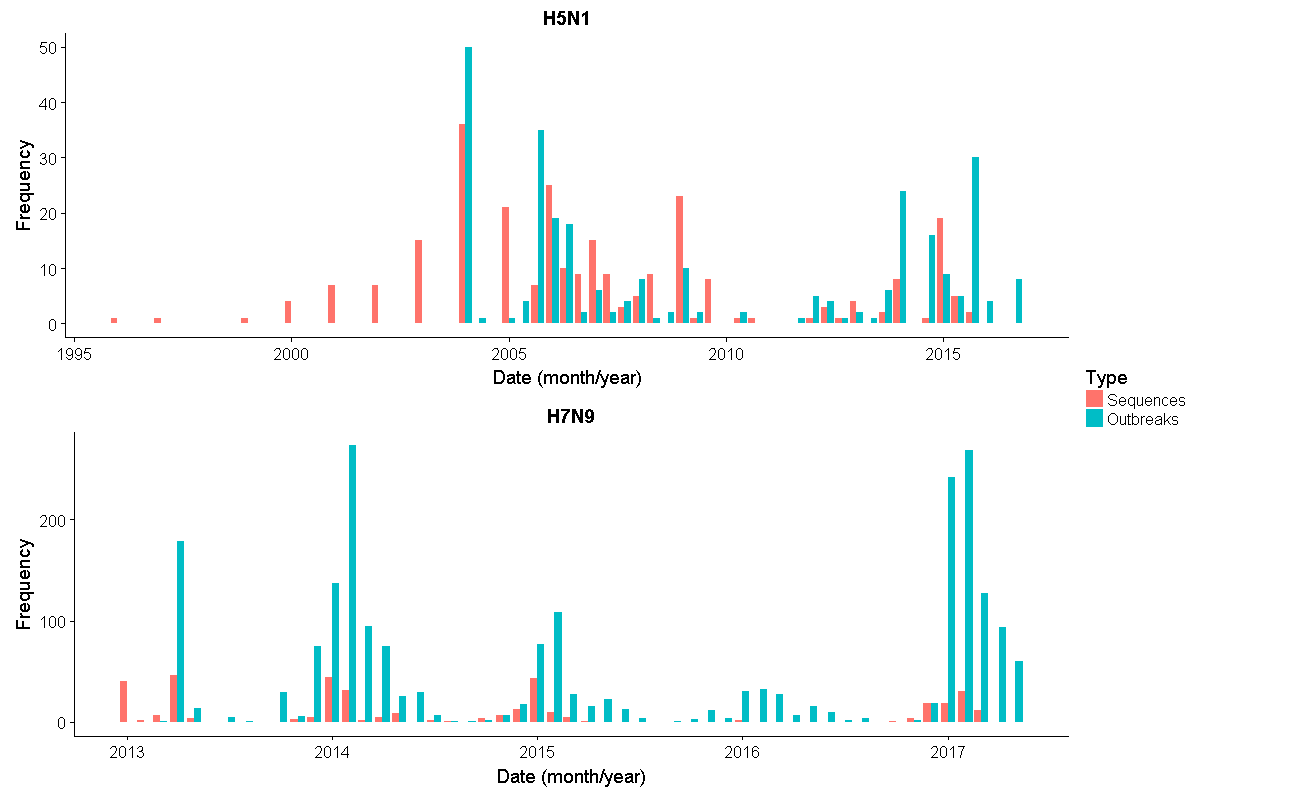
**

**Figure SF4. Comparison of H5N1 sequence and outbreak data over geographic space.** Orange and green colours represent sequence data and poultry outbreak/human case data respectively. Height of the bars are relative to each other. **
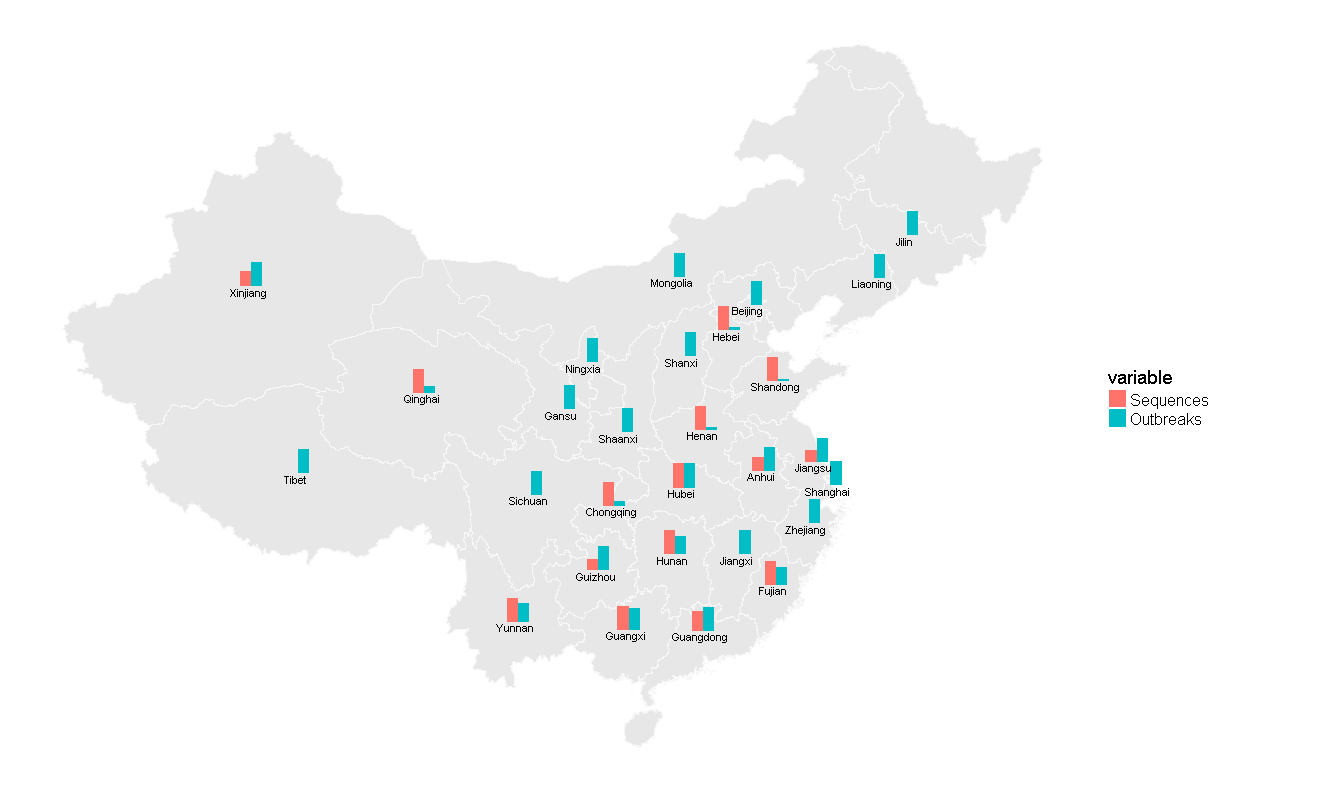
**

**Figure SF5. Comparison of H7N9 sequence and outbreak data over geographic space.** Orange and green colours represent sequence data and poultry outbreak/human case data respectively. Height of the bars are relative to each other.

**
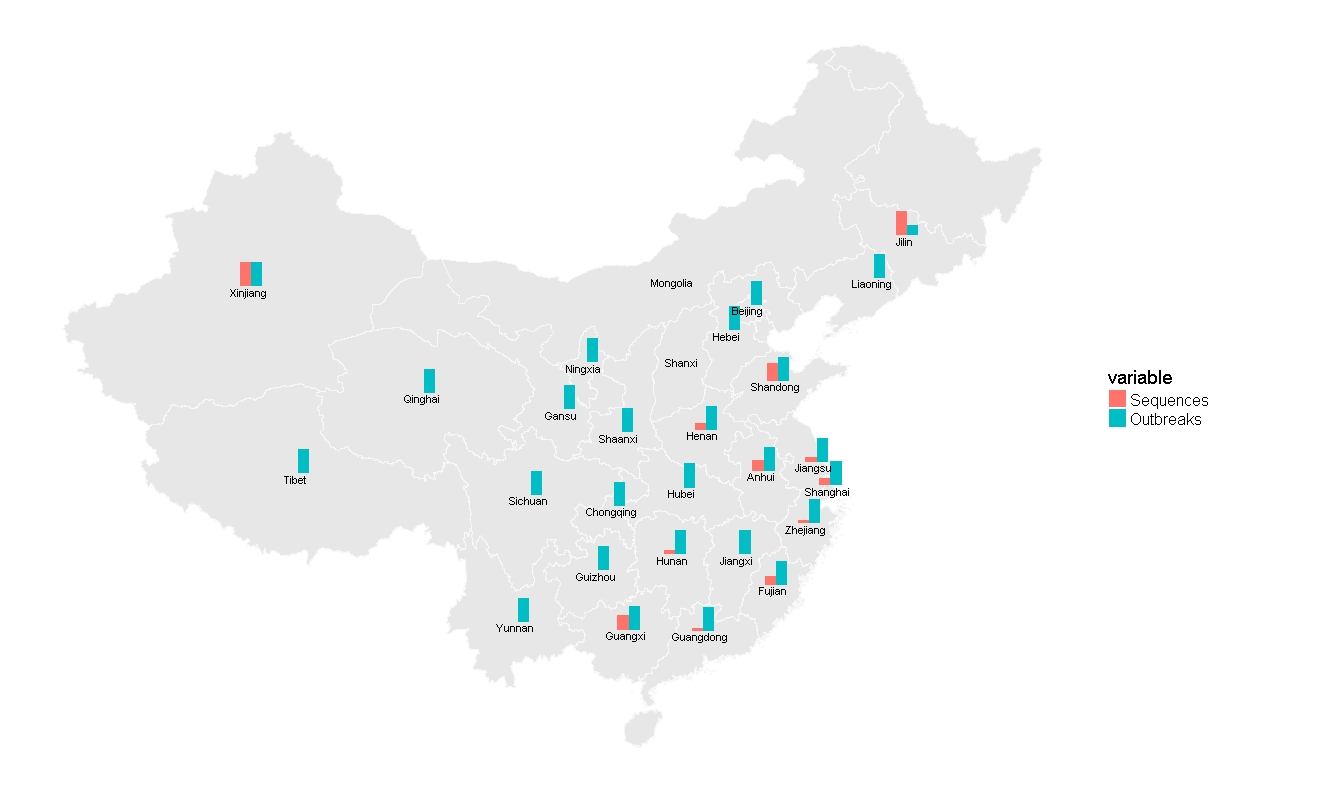
**

**Selection of GLM predictors**

Anthropogenic variables such as human population density and road densities have previously been identified as important contributors to predicting AI virus spatial distribution [^5-11^](#_ENREF_5). Highly populated areas are indicative of more intensive poultry farming practices, and highly travelled regions are indicative of more poultry trading activities. Likewise, agricultural variables indicative of the level of commercial poultry produced in an administrative region, have previously been identified as important contributors to predicting AI virus spatial distribution.

In addition to the agricultural variables chosen in [Lu, Leigh Brown ^12^](#_ENREF_12), we added H5N1 poultry vaccination rates as another variable. Previous studies have indicated selection pressures induced by avian influenza vaccination can significantly influence viral ecology in regions where vaccination occurs [^13^](#_ENREF_13). Also, the level of vaccination per province can be considered a proxy for the level of effort an administrative region invests in it’s animal health and biosecurity. Hence, H5N1 poultry vaccination rates per primary administrative region was included as a potential predictor (we were unable to include H7N9 poultry vaccination rates as, at the time of our analysis, these have only been recently rolled out in selected regions). The Chinese Ministry of Agriculture publishes monthly H5N1 vaccination rates of chickens, ducks and geese for each administrative region, however only chicken vaccination rates had high completion rates [^14^](#_ENREF_14). We collated data on the most recently published monthly vaccinations rates (from January to November 2014) for chickens, and averaged the rates for each administrative region.

Environmental variables have been previously demonstrated to have a role in modulating AIV distribution [^15^](#_ENREF_15)^,^ [^16^](#_ENREF_16). Relative humidity and precipitation have both been found to be major contributors in spatial and temporal models of H7N9 [^17-20^](#_ENREF_17). Changes in temperature and humidity have been shown to affect transmission potential [^21^](#_ENREF_21), for example ambient temperature may impact virus transmission due to physiological changes in the hosts which increase susceptibility to infections [^22^](#_ENREF_22).

Sequence sample size for each region was additionally incorporated as a predictor as this has previously been found to be a source of bias [^23^](#_ENREF_23). Similarly, the number of human cases and the number of poultry outbreaks can also be a potential source of bias. The total number of human cases and poultry outbreaks of H5N1 and H7N9 for each primary administrative region in China was obtained from the Food and Agricultural Organization (FAO) (last accessed May 2017) [^4^](#_ENREF_4). The average number of human cases and poultry outbreaks was calculated per year.

Finally, geographic distance between each of the regions was added as a predictor. Distance was calculated using centroid latitude and longitude coordinates derived from base maps of primary Chinese administrative regions (obtained from the GADM database of Global Administrative Areas [^24^](#_ENREF_24) in ArcMap version 10.2 under a China Albers Equal Area Conic projection [^25^](#_ENREF_25)).

In **Table SF1**, we list our 20 predictors selected for correlation analysis. **Supplementary Tables SF2-3** show the correlation results of the all potential predictors for H5N1 and H7N9 respectively. We provide the predictor data used in the GLM analysis in **Tables SF4-S5**.

**Table SF1 | Potential predictors collated for generalised linear model (GLM) analysis**

| **Category** | **Predictor name** | **Description** |
| --- | --- | --- |
| Anthropogenic | popDens | Population density (10, 000 persons/km^2^) |
|  | *ruralPop | Rural Population (%) |
|  | urbanPop | Urban Population (%) |
|  | *pollution | Sum of Smoke and Dust, Sulphur Dioxide, Nitrogen Oxides (10 000 tonnes) |
|  | railway | Freight Traffic by Region railway (10 000 tonnes) |
|  | highway | Freight Traffic by Region Highways (10 000 tonnes) |
|  | waterway | Freight Traffic by Region Waterways (10 000 tonnes) |
| Agricultural | *poultrySales | Sales of Poultry Per Capita Rural Household (kg) |
|  | outputEggs | Output of Poultry Eggs (10, 000 tonnes) |
|  | poultryDensity | Poultry Density (10, 000 unit/km^2^) |
|  | *Vaccination | Average 2014 monthly H5N1 vaccination rate (%) |
| Environmental | *natureReserves | Percentage of Nature Reserves in the Region (%) |
|  | temp | Average Temperature of Major Cities (^o^C) |
|  | *humidity | Average Relative Humidity of Major Cities (%) |
|  | precipitation | Average Precipitation of Major Cities (millimetres) |
|  | surfaceWater | Surface Water Resources (100 million cu.m) |
| Sampling bias | *sampleSize | Total number of sequences used in the analysis |
|  | Outbreaks | Average number of H5N1 or H7N9 domestic poultry outbreaks reported per year |
|  | Cases | Average number of H5N1 or H7N9 human cases reported per year |
| Geographical | *Distance | Distance between two locations, calculated using latitude and longitude coordinates |

*Predictor variables chosen for GLM analysis

**Table SF2 | Pearson correlation tests for potential predictors for H5N1.** Cells that are highlighted green indicate minimal correlation (R^2^ between -0.5 to 0.5). Cells that are highlighted orange indicate correlation is present (R^2^ > 0.5 or R^2^ < -0.5).

|  | poultrySales | outputEggs | poultryDensity | natureReserves | temp | humidity | precipitation | surfaceWater | pollution | urbanPop | ruralPop | popDens | railway | highway | waterway | sampleSize | outbreaks | cases |
| --- | --- | --- | --- | --- | --- | --- | --- | --- | --- | --- | --- | --- | --- | --- | --- | --- | --- | --- |
| Vaccination | 0.4 | 0.6 | 0.6 | -0.6 | 0.0 | -0.1 | 0.0 | -0.5 | 0.4 | 0.4 | -0.3 | 0.5 | 0.3 | 0.4 | 0.4 | -0.5 | -0.4 | -0.1 |
| poultrySales |  | 0.4 | 0.5 | -0.2 | 0.2 | -0.1 | 0.0 | -0.3 | 0.4 | 0.2 | -0.2 | 0.6 | 0.5 | 0.5 | 0.4 | -0.2 | -0.2 | 0.0 |
| outputEggs |  |  | 0.7 | -0.4 | -0.1 | -0.3 | -0.2 | -0.6 | 0.6 | 0.3 | -0.2 | 0.6 | 0.5 | 0.6 | 0.2 | -0.2 | -0.3 | -0.2 |
| poultryDensity |  |  |  | -0.4 | 0.1 | -0.1 | 0.0 | -0.4 | 0.5 | 0.2 | -0.2 | 0.8 | 0.5 | 0.7 | 0.4 | -0.2 | -0.3 | 0.0 |
| natureReserves |  |  |  |  | -0.1 | 0.0 | -0.1 | 0.3 | -0.3 | -0.2 | 0.1 | -0.4 | -0.4 | -0.4 | -0.4 | 0.6 | 0.3 | -0.1 |
| temp |  |  |  |  |  | 0.5 | 0.6 | 0.4 | -0.3 | 0.2 | -0.3 | 0.2 | -0.1 | 0.2 | 0.6 | 0.2 | 0.2 | 0.4 |
| humidity |  |  |  |  |  |  | 0.7 | 0.5 | -0.2 | 0.0 | -0.1 | 0.0 | -0.1 | -0.1 | 0.4 | 0.1 | 0.4 | 0.5 |
| precipitation |  |  |  |  |  |  |  | 0.4 | -0.3 | 0.2 | -0.2 | 0.1 | -0.3 | 0.0 | 0.4 | 0.1 | 0.3 | 0.5 |
| surfaceWater |  |  |  |  |  |  |  |  | -0.5 | -0.2 | 0.1 | -0.3 | -0.3 | -0.2 | 0.1 | 0.3 | 0.5 | 0.5 |
| pollution |  |  |  |  |  |  |  |  |  | 0.1 | 0.0 | 0.4 | 0.8 | 0.5 | 0.1 | -0.4 | 0.0 | -0.2 |
| urbanPop |  |  |  |  |  |  |  |  |  |  | -0.9 | 0.3 | 0.1 | 0.2 | 0.3 | -0.1 | -0.2 | -0.1 |
| ruralPop |  |  |  |  |  |  |  |  |  |  |  | -0.3 | 0.0 | -0.1 | -0.4 | 0.0 | 0.1 | 0.0 |
| popDens |  |  |  |  |  |  |  |  |  |  |  |  | 0.4 | 0.7 | 0.5 | -0.2 | -0.3 | 0.1 |
| railway |  |  |  |  |  |  |  |  |  |  |  |  |  | 0.6 | 0.2 | -0.4 | -0.2 | 0.0 |
| highway |  |  |  |  |  |  |  |  |  |  |  |  |  |  | 0.3 | -0.1 | -0.2 | 0.1 |
| waterway |  |  |  |  |  |  |  |  |  |  |  |  |  |  |  | -0.1 | 0.0 | 0.4 |
| sampleSize |  |  |  |  |  |  |  |  |  |  |  |  |  |  |  |  | 0.1 | 0.0 |
| outbreaks |  |  |  |  |  |  |  |  |  |  |  |  |  |  |  |  |  | 0.5 |

**Table SF3 | Pearson correlation tests for potential predictors for H7N9.** Cells are coloured based on a colour scale representing their R^2^ value. Cells that are highlighted green indicate minimal correlation (R^2^ between -0.5 to 0.5). Cells that are highlighted orange indicate correlation is present (R^2^ > 0.5 or R^2^ < -0.5).

|  | poultrySales | outputEggs | poultryDensity | natureReserves | temp | humidity | precipitation | surfaceWater | pollution | urbanPop | ruralPop | popDens | railway | highway | waterway | sampleSize | outbreaks | cases |
| --- | --- | --- | --- | --- | --- | --- | --- | --- | --- | --- | --- | --- | --- | --- | --- | --- | --- | --- |
| Vaccination | 0.4 | 0.3 | 0.3 | -0.4 | -0.2 | -0.2 | 0.0 | -0.5 | 0.0 | 0.4 | -0.4 | 0.4 | 0.0 | 0.1 | 0.2 | 0.1 | 0.1 | 0.1 |
| poultrySales |  | 0.1 | 0.3 | -0.2 | -0.1 | -0.1 | -0.1 | -0.3 | 0.1 | 0.3 | -0.3 | 0.4 | 0.3 | 0.1 | 0.2 | 0.1 | -0.1 | 0.1 |
| outputEggs |  |  | 0.4 | -0.1 | -0.4 | -0.4 | -0.4 | -0.5 | 0.6 | -0.2 | 0.2 | 0.2 | 0.6 | 0.5 | -0.1 | -0.2 | -0.2 | -0.1 |
| poultryDensity |  |  |  | -0.2 | -0.1 | -0.2 | -0.2 | -0.4 | 0.5 | 0.0 | 0.0 | 0.6 | 0.4 | 0.5 | 0.2 | -0.1 | 0.0 | 0.0 |
| natureReserves |  |  |  |  | -0.2 | -0.1 | -0.2 | 0.0 | 0.0 | -0.1 | 0.1 | -0.2 | 0.0 | -0.1 | -0.4 | -0.4 | -0.3 | -0.4 |
| temp |  |  |  |  |  | 0.8 | 0.7 | 0.5 | -0.3 | 0.2 | -0.2 | 0.0 | -0.2 | 0.1 | 0.4 | 0.5 | 0.6 | 0.5 |
| humidity |  |  |  |  |  |  | 0.8 | 0.6 | -0.2 | 0.2 | -0.2 | 0.0 | -0.1 | 0.1 | 0.3 | 0.5 | 0.4 | 0.4 |
| precipitation |  |  |  |  |  |  |  | 0.4 | -0.4 | 0.4 | -0.4 | 0.1 | -0.4 | -0.1 | 0.5 | 0.5 | 0.6 | 0.5 |
| surfaceWater |  |  |  |  |  |  |  |  | -0.2 | -0.1 | 0.1 | -0.4 | -0.2 | 0.1 | 0.1 | 0.3 | 0.2 | 0.2 |
| pollution |  |  |  |  |  |  |  |  |  | -0.2 | 0.2 | 0.3 | 0.6 | 0.6 | -0.1 | 0.0 | 0.0 | 0.0 |
| urbanPop |  |  |  |  |  |  |  |  |  |  | -1.0 | 0.4 | -0.3 | -0.2 | 0.5 | 0.3 | 0.4 | 0.4 |
| ruralPop |  |  |  |  |  |  |  |  |  |  |  | -0.4 | 0.3 | 0.2 | -0.5 | -0.3 | -0.4 | -0.4 |
| popDens |  |  |  |  |  |  |  |  |  |  |  |  | 0.1 | 0.3 | 0.5 | 0.2 | 0.2 | 0.3 |
| railway |  |  |  |  |  |  |  |  |  |  |  |  |  | 0.7 | -0.2 | -0.1 | -0.3 | -0.2 |
| highway |  |  |  |  |  |  |  |  |  |  |  |  |  |  | 0.0 | 0.1 | 0.0 | 0.1 |
| waterway |  |  |  |  |  |  |  |  |  |  |  |  |  |  |  | 0.8 | 0.6 | 0.8 |
| sampleSize |  |  |  |  |  |  |  |  |  |  |  |  |  |  |  |  | 0.5 | 0.8 |
| outbreaks |  |  |  |  |  |  |  |  |  |  |  |  |  |  |  |  |  | 0.7 |

**Table SF4 | Predictor values used in the H5N1 generalised linear model**

| **location** | **vaccination** | **poultrySales** | **natureReserves** | **humidity** | **pollution** | **ruralPop** | **sampleSize** |
| --- | --- | --- | --- | --- | --- | --- | --- |
| Anhui | 91.86 | 19.87 | 3.76 | 73.08 | 190.3 | 53.5 | 11 |
| Chongqing | 89.17 | 8.63 | 10.32 | 71.75 | 112.98 | 43.02 | 27 |
| Fujian | 92.02 | 3.77 | 3.11 | 75.17 | 109.11 | 40.4 | 11 |
| Guangdong | 87.05 | 10.81 | 6.73 | 81.58 | 243.09 | 32.6 | 21 |
| Guangxi | 80.65 | 7.77 | 5.98 | 79.75 | 130.21 | 56.47 | 20 |
| Guizhou | 87.84 | 2.45 | 5.41 | 84.58 | 189.91 | 63.59 | 9 |
| Hebei | 93.89 | 3.86 | 3.61 | 54.67 | 433.82 | 53.2 | 7 |
| Henan | 91.07 | 5.87 | 4.4 | 53.17 | 350.16 | 57.57 | 15 |
| Hubei | 91.69 | 2.8 | 5.14 | 81.42 | 161.21 | 46.5 | 16 |
| Hunan | 88.55 | 2.1 | 6.07 | 76.42 | 159.29 | 53.35 | 34 |
| Jiangsu | 95.85 | 8.73 | 4.1 | 68.08 | 291.48 | 37 | 7 |
| Qinghai | 79.49 | 0.08 | 30.21 | 59 | 43.64 | 52.56 | 32 |
| Shandong | 96.35 | 37.72 | 4.71 | 55.17 | 418.31 | 47.57 | 15 |
| Xinjiang | 85.23 | 4.27 | 12.95 | 53.08 | 231.17 | 56.02 | 14 |
| Yunnan | 84.08 | 2.21 | 7.45 | 66.83 | 160.71 | 60.69 | 26 |

**Table SF5 | Predictor values used in the H7N9 generalised linear model**

| **location** | **vaccination** | **poultrySales** | **natureReserves** | **humidity** | **pollution** | **ruralPop** | **sampleSize** |
| --- | --- | --- | --- | --- | --- | --- | --- |
| Anhui | 91.859 | 19.871 | 3.761 | 73.081 | 190.301 | 53.501 | 50.001 |
| Fujian | 92.02373 | 3.771 | 3.111 | 75.171 | 109.111 | 40.401 | 50.001 |
| Guangdong | 87.04736 | 10.811 | 6.731 | 81.581 | 243.091 | 32.601 | 50.001 |
| Guangxi | 80.64918 | 7.771 | 5.981 | 79.751 | 130.211 | 56.471 | 25.001 |
| Henan | 91.06918 | 5.871 | 4.401 | 53.171 | 350.161 | 57.571 | 10.001 |
| Hunan | 88.551 | 2.101 | 6.071 | 76.421 | 159.291 | 53.351 | 23.001 |
| Jiangsu | 95.84736 | 8.731 | 4.101 | 68.081 | 291.481 | 37.001 | 50.001 |
| Jilin | 91.71555 | 10.701 | 12.431 | 63.001 | 124.421 | 46.301 | 7.001 |
| Shandong | 96.35009 | 37.721 | 4.711 | 55.171 | 418.311 | 47.571 | 18.001 |
| Shanghai | 93.80211 | 23.331 | 5.221 | 69.501 | 71.691 | 10.701 | 24.001 |
| Xinjiang | 85.228 | 4.271 | 12.951 | 53.081 | 231.171 | 56.021 | 14.001 |
| Zhejiang | 91.97464 | 12.351 | 1.531 | 70.751 | 168.861 | 36.801 | 50.001 |

References

1. Tamura K, Dudley J, Nei M, Kumar S. MEGA4: Molecular Evolutionary Genetics Analysis (MEGA) software version 4.0. *Mol Biol Evol* 2007; **24**(8)**:** 1596-1599.

2. Robinson O, Dylus D, Dessimoz C. Phylo.io: Interactive Viewing and Comparison of Large Phylogenetic Trees on the Web. *Mol Biol Evol* 2016; **33**(8)**:** 2163-2166.

3. Jombart T, Kendall M, Almagro-Garcia J, Colijn C. treespace: Statistical exploration of landscapes of phylogenetic trees. *Molecular ecology resources* 2017; **17**(6)**:** 1385-1392.

4. FAO FaAO. *EMPRES-i Global Animal Disease Information System* 2017. Available from: <http://EMPRES-i.fao.org/> (accessed May 2017)

5. Li XH, Tian HD, Heiner M, Li DM. Global Occurrence and Spread of Highly Pathogenic Avian Influenza Virus of the Subtype H5N1. *Avian Dis* 2011; **55**(1)**:** 21-28.

6. Adhikari D, Chettri A, Barik SK. Modelling the ecology and distribution of highly pathogenic avian influenza (H5N1) in the Indian subcontinent. *Curr Sci* 2009; **97**(1)**:** 73-78.

7. Pfeiffer DU, Minh PQ, Martin V, Epprecht M, Otte MJ. An analysis of the spatial and temporal patterns of highly pathogenic avian influenza occurrence in Vietnam using national surveillance data. *The Veterinary Journal* 2007; **174**(2)**:** 302-309.

8. Gilbert M, Xiao X, Pfeiffer DU, Epprecht M, Boles S, Czarnecki C*, et al.* Mapping H5N1 highly pathogenic avian influenza risk in Southeast Asia. *Proceedings of the National Academy of Sciences* 2008; **105**(12)**:** 4769-4774.

9. Ward MP, Maftei D, Apostu C, Suru A. Environmental and anthropogenic risk factors for highly pathogenic avian influenza subtype H5N1 outbreaks in Romania, 2005--2006. *Vet Res Commun* 2008; **32**(8)**:** 627-634.

10. Gilbert M, Xiao X, Chaitaweesub P, Kalpravidh W, Premashthira S, Boles S*, et al.* Avian influenza, domestic ducks and rice agriculture in Thailand. *Agric Ecosyst Environ* 2007; **119:** 409-415.

11. Fang L-Q, de Vlas SJ, Liang S, Looman CWN, Gong P, Xu B*, et al.* Environmental Factors Contributing to the Spread of H5N1 Avian Influenza in Mainland China (Avian Influenza). *PLoS ONE* 2008; **3**(5)**:** e2268.

12. Lu L, Leigh Brown AJ, Lycett SJ. Quantifying predictors for the spatial diffusion of avian influenza virus in China. *BMC Evol Biol* 2017; **17**(1)**:** 16.

13. Cattoli G, Fusaro A, Monne I, Coven F, Joannis T, El-Hamid HS*, et al.* Evidence for differing evolutionary dynamics of A/H5N1 viruses among countries applying or not applying avian influenza vaccination in poultry. *Vaccine* 2011; **29**(50)**:** 9368-9375.

14. Chinese Ministry of Agriculture. *Veterinary Bureau, Surveillance and Outbreak, H5N1 bird flu and foot-and-mouth disease surveillance information*. 2017. Available from: <http://www.syj.moa.gov.cn/dwyqdt/jcxx/> (accessed 1 Oct 2017)

15. Tuncer N, Martcheva M. Modeling Seasonality in Avian Influenza H5n1. *Journal of Biological Systems* 2013; **21**(4)**:** 1340004.

16. Zhang Y, Feng C, Ma C, Yang P, Tang S, Lau A*, et al.* The impact of temperature and humidity measures on influenza A (H7N9) outbreaks-evidence from China. *International journal of infectious diseases : IJID : official publication of the International Society for Infectious Diseases* 2015; **30:** 122-124.

17. Li XL, Yang Y, Sun Y, Chen WJ, Sun RX, Liu K*, et al.* Risk Distribution of Human Infections with Avian Influenza H7N9 and H5N1 virus in China. *Sci Rep* 2015; **5:** 18610.

18. Fang L-Q, Li X-L, Liu K, Li Y-J, Yao H-W, Liang S*, et al.* Mapping Spread and Risk of Avian Influenza A (H7N9) in China. *Sci Rep* 2013; **3:** Article No. 2722.

19. Hu W, Zhang W, Huang X, Clements A, Mengersen K, Tong S. Weather variability and influenza A (H7N9) transmission in Shanghai, China: A Bayesian spatial analysis. *Environ Res* 2015; **136**(0)**:** 405-412.

20. Zhang Y, Feng C, Ma C, Yang P, Tang S, Lau A*, et al.* The impact of temperature and humidity measures on influenza A (H7N9) outbreaks-evidence from China. *Int J Infect Dis* 2015; **30:** 122-124.

21. Lowen AC, Steel J. Roles of humidity and temperature in shaping influenza seasonality. *J Virol* 2014; **88**(14)**:** 7692-7695.

22. Dowell SF, Ho MS. Seasonality of infectious diseases and severe acute respiratory syndrome–what we don't know can hurt us. *The Lancet Infectious Diseases* 2004; **4**(11)**:** 704-708.

23. Magee D, Suchard MA, Scotch M. Bayesian phylogeography of influenza A/H3N2 for the 2014-15 season in the United States using three frameworks of ancestral state reconstruction. *PLoS Comput Biol* 2017; **13**(2)**:** e1005389.

24. *GADM database of Global Administrative Areas* 2015. Available from: <http://gadm.org/> (accessed May 20 2017)

25. Australian Centre for Asian Spatial Information and Analysis Network (ACASIAN). *China Albers Equal Area Conic*. Available from: <http://spatialreference.org/ref/sr-org/7564/> (accessed 2017)
