# Supplementary material for: Characterising routes of H5N1 and H7N9 spread in China using Bayesian phylogeographical analysis
Source: Emerg Microbes Infect. 2018 Nov 21;7:184. doi: 10.1038/s41426-018-0185-z (PMC6246557; doi:10.1038/s41426-018-0185-z)
Supplement: Supplementary file 9 — Supplementary Tables [file 41426_2018_185_MOESM9_ESM.docx]

SUPPLEMENTARY TABLES

**Table S1 | Interpretation of computed Bayes Factor values**

| Bayes Factor (BF) | Interpretation |
| --- | --- |
| >100 | Definitive support |
| 30-100 | Very strongly support |
| 10-30 | Strongly support |
| 6-10 | Support |
| 3-6 | Marginal support |

**Table S2 | Summary of phylogenetic generalised linear model results for H5N1**

| **Predictor** | **Type** | **Inclusion Probabilities**§ | **Bayes Factor** | **Coefficient (95% CI)** † |
| --- | --- | --- | --- | --- |
| poultrySales | Destination | < 0.01 | 0.03 | 0.14 (-0.07 to 0.51) |
| pollution | Destination | < 0.01 | 0.04 | 0.05 (-0.23 to 0.4) |
| vaccination | Destination | < 0.01 | 0.06 | -0.15 (-0.39 to 0.09) |
| poultrySales | Origin | < 0.01 | 0.06 | 0.49 (-0.77 to 1.48) |
| ruralPop | Destination | < 0.01 | 0.06 | 0.15 (-0.03 to 0.32) |
| natureReserves | Destination | < 0.01 | 0.08 | 0.03 (-0.5 to 0.46) |
| vaccination | Origin | < 0.01 | 0.09 | 0.49 (-0.25 to 1.43) |
| natureReserves | Origin | < 0.01 | 0.10 | -0.1 (-1.15 to 1.52) |
| humidity | Origin | < 0.01 | 0.11 | 0.26 (-0.39 to 0.96) |
| sampleSize | Destination | 0.01 | 0.29 | 0.27 (0.05 to 0.56) |
| humidity | Destination | 0.01 | 0.39 | -0.42 (-0.93 to -0.06) |
| pollution | Origin | 0.05 | 1.98 | 1.04 (0.2 to 1.93) |
| sampleSize | Origin | 0.07 | 2.51 | 1.21 (0.23 to 2.43) |
| *ruralPop | Origin | 0.94 | >100 | -0.67 (-1 to -0.34) |
| *Distance | NA | 1.00 | >100 | -0.74 (-1.02 to -0.46) |

* Top two strongest predictors in the H7N9 generalized linear model

†Mean coefficient and 95% highest posterior density credible interval (CI)

§Probability that the predictor was included in the model

**Table S3 | Summary of phylogenetic generalised linear model results for H7N9**

| **Predictor** | **Type** | **Inclusion Probabilities**§ | **Bayes Factor** | **Coefficient (95% CI)** † |
| --- | --- | --- | --- | --- |
| poultrySales | Destination | < 0.01 | 0.08 | 0.02 (-0.47 to 0.75) |
| pollution | Destination | < 0.01 | 0.08 | 0.3 (-0.66 to 1.15) |
| poultrySales | Origin | 0.02 | 0.58 | -1.36 (-2.86 to 0.53) |
| humidity | Origin | 0.02 | 0.89 | 0.91 (-1.94 to 3.17) |
| vaccination | Origin | 0.03 | 0.98 | 0.78 (-0.49 to 1.91) |
| ruralPop | Destination | 0.04 | 1.64 | 1.27 (0.19 to 2.87) |
| ruralPop | Origin | 0.07 | 2.82 | -0.6 (-2.55 to -0.18) |
| pollution | Origin | 0.08 | 2.95 | 1.8 (0.15 to 4.02) |
| vaccination | Destination | 0.12 | 4.89 | -0.8 (-1.29 to -0.33) |
| sampleSize | Origin | 0.17 | 7.34 | 1.93 (0.4 to 4.59) |
| natureReserves | Destination | 0.18 | 7.61 | -0.99 (-1.96 to -0.33) |
| humidity | Destination | 0.25 | 12.18 | 1.8 (0.56 to 3.67) |
| sampleSize | Destination | 0.26 | 12.80 | 0.84 (0.34 to 1.67) |
| *natureReserves | Origin | 0.34 | 18.75 | -1.18 (-1.9 to -0.47) |
| *Distance | NA | 0.94 | >100 | -0.82 (-1.16 to -0.47) |

* Top two strongest predictors in the H7N9 generalized linear model

†Mean coefficient and 95% highest posterior density credible interval (CI)

§Probability that the predictor was included in the model

**Table S4 | Number of H5N1 and H7N9 sequences per discrete location**

| **Virus** | **Location** | **Freq** |
| --- | --- | --- |
| *H5N1* | Anhui | 11 |
|  | Chongqing | 27 |
|  | Fujian | 11 |
|  | Guangdong | 21 |
|  | Guangxi | 20 |
|  | Guizhou | 9 |
|  | Hebei | 7 |
|  | Henan | 15 |
|  | Hubei | 16 |
|  | Hunan | 34 |
|  | Jiangsu | 7 |
|  | Qinghai | 32 |
|  | Shandong | 15 |
|  | Xinjiang | 14 |
|  | Yunnan | 26 |
| *H7N9* | Anhui | 50 |
|  | Fujian | 50 |
|  | Guangdong | 50 |
|  | Guangxi | 25 |
|  | Henan | 10 |
|  | Hunan | 23 |
|  | Jiangsu | 50 |
|  | Jilin | 7 |
|  | Shandong | 18 |
|  | Shanghai | 24 |
|  | Xinjiang | 14 |
|  | Zhejiang | 50 |
